# An Age

**Published:** 1883-12

**Authors:** 


// HALL'S
JOURNAL OF HEALTH
AND
MISCELLANY.

MONTHLY.

E. H. GIBBS, A. M., M. D., EDITOR.

FOUNDED IN 1854.

### AN AGE.

THIS number of the JOURNAL for December, 1883, completes the thirtieth year of its existence. Thirty years have wrought marvelous changes in the affairs of the world. A generation of men has disappeared forever, and others have taken their places.

The world has advanced more in the past thirty years than during the previous century; not only in the arts and sciences, but in health and physical well-being, because of the increase and general diffusion of knowledge of sanitary laws. The average term of human life has thus been considerably extended.

The average term of life of the nobility of England is sixty years, or about double that of the masses of the people. There are other classes in England and every other civilized country whose average term of life would come up to sixty years; they are men who are able to surround themselves with the comforts of life, and have the intelligence to enjoy them rationally, and the wisdom to protect themselves against evils and vicissitudes that tend to weaken and destroy. The human race illustrates the theory of the "survival of the fittest"; still it is a most discouraging fact for the philanthropist that men seemingly intelligent in business affairs are often too ignorant of matters that are essential to health to protect themselves and their families against diseases that lurk in their home and its surroundings, but are concealed from *their* eyes.

There is a class of diseases that almost always originates in contamination of drinking water; they are notably diphtheria and typhoid fever. Almost every village is periodically scourged with these diseases, and farm-houses, however secluded, by no means escape. All the children of a family, in many cases, fall victims to one or both of these diseases, and certainly through the ignorance or neglect of those who are responsible for the existence of those conditions that produce diseases. Such ignorance is criminal. There should be a competent sanitary inspector in every county in the United States, whose duty it should be to test the water supply of every house, and with authority to enforce such changes and regulations as he might deem necessary to the health of the community in respect to other things also.

